# Novel Decision Tool for More Severe α-Thalassemia Genotypes Screening with Functional Loss of Two or More α-Globin Genes: A Diagnostic Test Study

**DOI:** 10.3390/diagnostics12123008

**Published:** 2022-12-01

**Authors:** Patricia F. R. Siqueira, Marcos K. Fleury, Robéria M. Pontes, Renata S. P. Silva, Elaine S. Costa, Marcelo G. P. Land

**Affiliations:** 1 Multidisciplinary Laboratory, Instituto de Puericultura e Pediatria Martagão Gesteira (IPPMG), Faculty of Medicine, Federal University of Rio de Janeiro (UFRJ), Rio de Janeiro 21941-912, Brazil; 2 Internal Medicine Postgraduate Program, Faculty of Medicine, Federal University of Rio de Janeiro (UFRJ), Rio de Janeiro 21941-913, Brazil; 3 Department of Clinical and Toxicological Analysis, Faculty of Pharmacy, Federal University of Rio de Janeiro (UFRJ), Rio de Janeiro 21941-599, Brazil; 4 Translational Research Laboratory, Children’s Hospital of Brasilia José Alencar, Brasilia 70684-831, Brazil; 5 Hematology Department, Instituto de Puericultura e Pediatria Martagão Gesteira (IPPMG), Faculty of Medicine, Federal University of Rio de Janeiro (UFRJ), Rio de Janeiro 21941-912, Brazil

**Keywords:** α-thalassemia, microcytic anemia, molecular diagnosis, binomial logistic regression, prediction, ROC curve, sensitivity, specificity

## Abstract

After the exclusion of iron deficiency and β-thalassemia, molecular research for α-thalassemia is recommended to investigate microcytic anemia. Aiming to suggest more efficiently the molecular analysis for individuals with a greater chance of having a symptomatic form of the disease, we have developed and validated a new decision tool to predict the presence of two or more deletions of α-thalassemia, increasing considerably the pre-test probability. The model was created using the variables: the percentage of HbA_2_, serum ferritin and mean corpuscular volume standardized by age. The model was trained in 134 patients and validated in 160 randomly selected patients from the total sample. We used Youden’s index applied to the ROC curve methodology to establish the optimal odds ratio (OR) cut-off for the presence of two or more α-globin gene deletions. Using the OR cut-off of 0.4, the model’s negative predictive value (NPV) was 96.8%; the cut-off point accuracy was 85.4%; and the molecular analysis pre-test probability increased from 25.9% to 65.4% after the use of the proposed model. This tool aims to assist the physician in deciding when to perform molecular studies for the diagnosis of α-thalassemia. The model is useful in places with few financial health resources.

## 1. Introduction

The molecular basis of α-thalassemia is related to a reduction (α^+^-thalassemia) or absence (α^0^-thalassemia) from the synthesis of α-globin chains. The phenotypic differences in this disease are due to the number of α-globin genes affected by some mutation. In general, these mutations are caused by deletions of one or both α-globin genes from each chromosome 16 [1,2]. Its prevalence varies considerably around the world, occurring in high frequency throughout tropical and subtropical regions [2,3]. The estimated prevalence of α^+^-thalassemia is at least 20% of the world population [4]. The prevalence of α-thalassemia carriers in Brazil is not fully known. We can attribute this to the great territorial dimensions of the country and the variability of the genetic constitution of its population, in addition to the lack of availability of molecular testing for population testing in many places. It is known, however, that the -α^3.7^ deletion in heterozygosis, or homozygosis, is the most prevalent, due to the great miscegenation of the population with important African genetic contributions [5,6,7]. Studies in several Brazilian regions report variable frequencies of α^+^-thalassemia resulting from the -α^3.7^ deletion, ranging from 0.7 to 20% [5,6,8,9,10,11]. After screening for the presence of microcytosis and/or hypochromia, the -α^3.7^ deletion is detected in around 50% of individuals [12,13]. The detection of these deletions is usually performed by multiplex polymerase chain reaction (multiplex-PCR) [3,14]. Other molecular techniques, such as multiplex ligation-dependent probe amplification (MLPA), gene sequencing, or amplification refractory mutations (ARMS), may be required for the investigation of unknown deletions or point mutations in α-globin genes [15].

In routine medical practice, laboratory tests exclude other causes of microcytic and/or hypochromic anemia before investigating major α-thalassemia deletions. For the initial investigation, it is recommended to study the iron kinetics, hematimetric indices, peripheral blood smears analysis, detection of H hemoglobin (HbH) inclusions, reticulocyte count, and analysis of the hemoglobin profile [2,16,17]. In the presence of microcytosis and/or hypochromia, after excluding iron deficiency and β-thalassemia minor, molecular tests for α-thalassemia by PCR are recommended [18,19]. However, the availability of molecular analysis of patients in primary care hospitals is not a reality in developing or underdeveloped countries. For this reason, using inexpensive and efficient screening methods to select individuals who require molecular testing for α-thalassemia can reduce health system costs.

In the present study, we developed and validated a binomial logistic regression (BLR) model using the results of laboratory tests, usually applied as screening, to decide when to perform the molecular technique. The use of this model aims to increase the molecular analysis pre-test probability of selecting patients before performing multiplex-PCR for the main deletions in the α-globin gene. Thus, the number of patients tested by PCR can considerably decrease, generating significant cost savings and reducing the need for blood sampling, which is especially important in infancy.

For this purpose, we performed the detection of the deletions -α^3.7^ and -α^4.2^ in 294 patients who underwent analysis for investigation of α-thalassemia at the Clinical Analysis Laboratory of Faculty of Pharmacy (LACFAR) at the Federal University of Rio de Janeiro (UFRJ). Four of these patients presented a characteristic peak of HbH in HPLC, in addition to HbH in erythrocytes staining with brilliant cresyl blue. The deletions -(α)^20.5^, --^SEA,^, and --^MED^ were also tested in these patients. 

## 2. Materials and Methods

This study was previously approved by the Ethics Committee of the IPPMG hospital (Comitê de Ética em Pesquisa do Instituto de Puericultura e Pediatria Martagão Gesteira—Federal University of Rio de Janeiro). The first acceptance (Acceptance Number 38/11, Approval Date: 14 June 2011) was granted to evaluate the prevalence of α-thalassemia in children with microcytosis in a pediatric hospital in Rio de Janeiro. For the construction and validation of the mathematical model, we requested approval from the same Ethics Committee to use the data of other patients evaluated for α-thalassemia in the Clinical Analysis Laboratory of the Faculty of Pharmacy at the Federal University of Rio de Janeiro (UFRJ) (Acceptance Number 847.419—Approval Date: 28 October 2014). 

### 2.1. Analytical Methods

This was a retrospective observational study of a cohort of patients (adults, adolescents, and children) from November 2010 to July 2015. Peripheral blood samples of 294 patients presenting microcytosis, or suspected of being α-thalassemia carriers, were submitted to PCR for detection of the deletions -α^3.7^ and -α^4.2^ for the investigation of α^+^-thalassemia, as well as for a complete blood count (CBC), high-performance liquid chromatography (HPLC), reticulocyte count, research of HbH inclusions in erythrocytes, measurements of iron status: serum iron, total iron-binding capacity (TIBC), serum ferritin, transferrin saturation (TS), and biochemical measurements such as lactate dehydrogenase (LDH), direct bilirubin (DBil), indirect bilirubin (IBil), and total bilirubin (TBil). Four patients with HbH inclusions in their erythrocytes and the HbH characteristic peak in the HPLC were also tested for other deletions, --^MED^, --^SEA^, and -(α)^20.5^, related to α^0^-thalassemia. 

These results were recorded in a database associated with the following variables: age (years), gender, number of α-globin genes deleted, CBC including red cell count (RCC), hemoglobin (Hb), hematocrit (Ht), mean corpuscular volume (MCV), MCV standardized by age, mean corpuscular hemoglobin (MCH), mean corpuscular hemoglobin concentration (MCHC), red blood cell distribution width (RDW), percentage of Hb A_1_, Hb A_2_, and fetal Hb, serum iron, serum ferritin, TIBC, TS, LDH, DBil, IBil, and TBil. 

The CBC was performed on an automated cell counter, the ABX Pentra 60C+ (Horiba, Montpellier, France). The serum ferritin levels were determined by an enzyme immunoassay with the Unicel DxI 600 (Beckman Coulter, Brea, CA, USA). The blood of all the patients was analyzed by cation-exchange high-performance liquid chromatography (HPLC Variant, Bio-Rad Laboratories, Hercules, CA, USA) for the quantification of hemoglobin (Hb) A_1_, A_2_, and fetal and variant hemoglobin, when identified by the methodology. All the samples were analyzed for the presence of HbH inclusion bodies and the reticulocyte count after staining the peripheral blood cells with 1% brilliant cresyl blue for 1 h at 37 °C. The other biochemical measurements and iron status were performed but were not used to build the BLR model.

The genomic DNA was isolated from the peripheral blood leucocytes using a Biopur Mini Spin Plus kit (Biometrix Diagnóstica, Curitiba, Brazil). In all the peripheral blood samples, the molecular characterization of α-thalassemia was determined by multiplex polymerase chain reaction (multiplex-PCR) using previously designed primers to detect the deletions -α^3.7^, -α^4.2^ and the not deleted α2 gene [3]. Each set of reactions included positive and negative controls. Furthermore, the deletions -(α)^20.5^, --^SEA^, and --^MED^ were also investigated in four patients who presented with HbH in the erythrocytes staining with brilliant cresyl blue, associated with the characteristic peak of HbH in the HPLC. 

Each 25 μL reaction contained 20 mmol/L Tris-HCl (pH 8.4), 50 mmol/L KCl, 2.0 mmol/L MgCl_2_, 1,0 mol/L betaine (Sigma-Aldrich, St. Louis, MO, USA), 0.5 mmol/L of each dNTP, 15 pmoles of each specific primer for the deletions -α^3.7^, -α^4.2^ and the not deleted α2 gene, 2.5 units of Taq DNA Polymerase (Invitrogen, Life Technologies, São Paulo, Brazil), and 100 ng of genomic DNA. When the multiplex-PCR was performed for the investigation of other deletions (--^MED^, --^SEA^, and -(α)^20.5^), 5 pmoles of each specific primer were added in this reaction, maintaining the final reaction volume of 25 µL. The reactions were carried out on a thermal cycler Veriti (Applied Biosystems, Foster City, CA, USA) with an initial denaturation at 95 °C for 5 min, 30 cycles of 98 °C for 45 s, 63 °C for 1 min 30 s, 72 °C for 2 min 15 s, and a final extension at 72 °C for 5 min. After amplification, 15 μL of the product was electrophoresed through a 1% agarose gel in 1 X TBE for 1 h. The agarose gel was stained with ethidium bromide and visualized on an ultraviolet transilluminator. 

Due to the large variation of the reference values of the MCV related to patient age, we created a standardization of MCV for all patients relative to the minimum reference value for adults (80 fL) [13]. This strategy permitted the MCV to be homogeneously analyzed in the different age ranges studied. The MCV minimum expected values for the age were previously described [16]. The normal values of the MCV standardized by age were the same as those used for the adult population (80–100 fL). 

The following formula was used to calculate the MCV standardized by age:

$$\mathrm{MCV}\;\mathrm{standardized}\;\mathrm{by}\;\mathrm{age}=\frac{\mathrm{MCV}\;\mathrm{observed}}{\mathrm{MCV}\;\mathrm{minimum}\;\mathrm{expected}\;\mathrm{for}\;\mathrm{the}\;\mathrm{age}}\times 80$$

### 2.2. Binomial Logistic Regression (BLR) Model Building 

The statistical analyses were performed using the Statistical Package for the Social Sciences (SPSS) software program, version 20.0, SPSS Inc., Chicago, IL, USA. 

The group of 294 patients used for the construction and validation of the BLR model was named the total sample. The training set consisted of 134 individuals who were selected randomly by the SPSS software from the data of the 294 patients. 

The univariate and multivariate analyses were performed with the training set to identify the most significant variables for the development of the BLR model. 

Several variables were evaluated using univariate analysis. The variables with *p*-value ≤ 0.2 were selected for the multivariate analysis. We first evaluated the collinearity between the variables, using the Pearson correlation coefficient, Spearman correlation coefficient, and Kendall’s Tau test. The candidate variables were included in a full model. The parsimonious model was built through a process of backward elimination using the likelihood-ratio test. This final model included the serum ferritin, MCV standardized by age and percentage of Hb A_2_.

A receiver operating characteristic (ROC) analysis was used to determine the optimal odds ratio (OR) cut-off point to predict the presence of two or more deletions related to α-thalassemia. Based on Youden’s index applied to the ROC curve, the cut-off (OR > 0.4 or probability > 28.6%) achieved the highest possible sensitivity and specificity. The same cut-off was used for the analysis of the validation group. 

The validation set (n = 160) was composed of other randomly selected individuals from the total sample. The performance of the BLR model in both sets was also evaluated in terms of the sensitivity, specificity, positive predictive value (PPV), negative predictive value (NPV), positive likelihood ratio and negative likelihood ratio, the area under the curve (AUC), and cut-off point accuracy. 

## 3. Results

### 3.1. Univariate Analysis

The variables evaluated by the univariate analysis are reported in Table 1. The variables with a *p*-value < 0.2 appear in bold. 

### 3.2. Development of BLR Model 

The variables included in the final parsimonious model were as follows: the MCV standardized by age, serum ferritin, and percentage of Hb A_2_. The parameters of the multivariate analysis of these variables are shown in Table 2.

The resulting BLR model after fitting to the training data can be expressed as the OR and as a probability. Table 3 shows how the value of OR and probability are calculated using the proposed model.

### 3.3. Determination of the Best Cut-Off Point and Its Performance

To determine the best cut-off point to the OR to predict the outcome, we used the ROC curve. The optimal cut-off point to predict two or more α-globin gene deletions was OR > 0.4.

**Figure 1:**
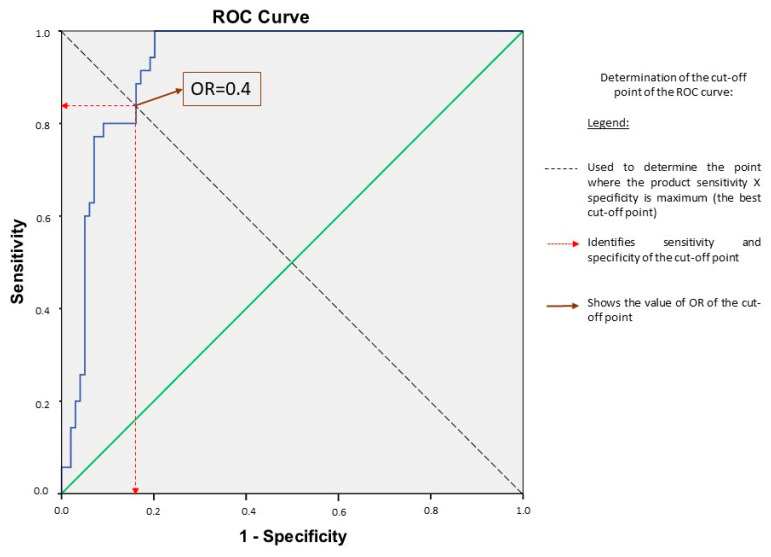
Determination of the optimal cut-off point using the ROC curve.

We used the OR > 0.4 cut-off point to create the predicted group variable. We compared the predicted group with the gold standard (two or more α-globin gene deletions detected by multiplex-PCR). Figure 1 shows that the BLR model gave an AUC of 0.927 with an accuracy of the cut-off point of 85.1% when setting the cut-off point at OR > 0.4 (or probability >28.6%). We compared the predicted group with the gold standard (two or more α-globin gene deletions detected by multiplex-PCR). This model performed a sensitivity of 88.6%, specificity of 83.8%, PPV of 66.0%, and NPV of 95.4% (Supplementary Table S1).

### 3.4. Validation Process

Table 4 shows the genotypic characteristics, the age distribution, and the variables associated with the BLR model in both groups (training and validation data sets). There was no significant difference between the groups. The variables of the BLR model and age were analyzed using the Mann–Whitney test or Student’s *t*-test. The comparison between the genotypes of the α-globin gene of the two groups and sex was performed using the chi-square test or Fisher’s exact test.

The model validation was performed on another 160 patients randomly selected from the total sample, using the OR > 0.4 cut-off point to create the predicted group variable. We compare the predicted group with the gold standard (two or more α-globin gene deletions detected by multiplex-PCR). This model gave an AUC of 0.902 with an accuracy of the cut-off point of 85.6% using the same probability cut-off of 28.6% for the validation of the training set. This model performed a sensitivity of 95.1%, specificity of 82.4%, PPV of 65.0%, and NPV of 98.0% (Supplementary Table S2). 

Applying the same cut-off point in the total sample (n = 294), results were obtained that were very similar to those of the training and validation sets, which demonstrates the good randomization of the cohort, as can be seen in Table 5 (Supplementary Table S3).

### 3.5. Improving the Decision-Making Process 

The pre-test probability, without using the model, calculated by the percentage of positive patients for two or more deletions tested by PCR, was 26.1% (35/134) in the training, 25.6% (41/160) in the validation set, and 25.9% (76/294) in the total sample. After the application of the BLR model (selecting molecular analysis only for the patients considered positive), the probability pre-test increased to 66.0% (31/47), 65.0% (39/60), and 65.4% (70/107), respectively. In the model established, the percentage of false negatives was 1.3–3.0%. 

**Figure 2:**
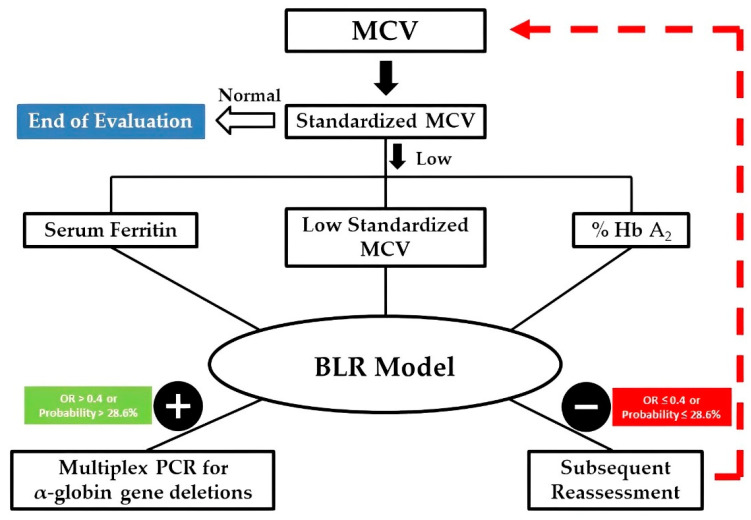
Algorithm to use the BRL model for investigation of α-thalassemia.

Using our model, about two-thirds of patients with suspected α-thalassemia would not need multiplex-PCR. We propose an algorithm for the evaluation of all patients suspected of being α-thalassemia carriers, to be applied in clinical practice (Supplementary Excel worksheet S4). If the result of the model was negative, the patient can be reassessed at another time, maybe a year later, if the suspicion persists, as shown in Figure 2.

## 4. Discussion

Several algorithms based on RBC indices have been proposed to aid in the differential diagnosis of iron deficiency anemia (IDA) and thalassemia traits. [20,21,22,23,24]. Most of them were constructed to distinguish IDA from β-thalassemia [24,25,26,27]. An exception is the Huber-Herklotz index, developed to differentiate cases of IDA from α-thalassemia using the parameters of complete blood cell counts [28,29,30]. Recently, some groups have developed models that allow the distinction of α-thalassemia from β-thalassemia and iron deficiency. In China, a model was described associating some indices of red blood cells with Hb A_2_ levels to differentiate β-thalassemia, α-thalassemia, and iron deficiency [31]. An algorithm has been developed to differentiate between α-thalassemia and β-thalassemia traits (αβ-algorithm), especially in geographic regions with large numbers of individuals with these inherited blood disorders [32].

The diagnosis of thalassemia traits requires time and resources, with the performance of several screenings and specific exams. Beta thalassemia is usually identified in the laboratory by the observation of Hb A_2_ greater than 3.5%, although, sometimes, when there is an association with iron deficiency or coinheritance with α- or δ-thalassemia or depending on the type of mutation in the β-globin gene, the Hb A_2_ can be detected in the normal concentration [15,31]. On the other hand, the definitive diagnosis of α-thalassemia is only possible using molecular techniques to identify α-globin gene mutations, in addition to other screening tests.

The most frequent α-thalassemia-related mutations detected in the Brazilian population are the -α^3.7^ and -α^4.2^ deletions, due to the African heritage, which is very present in this population [5,7,9,10,33,34,35]. These mutations, when in heterozygosity, affect one α-gene, making this individual a silent carrier who is asymptomatic and does not need treatment. To optimize molecular testing by identifying the patients with a higher chance of having two or more α-globin genes mutated, we created this model. This is the first study to develop and validate a BLR model to predict the OR that a patient has two or more deletions related to α-thalassemia. 

We know that, under ideal conditions, molecular testing for α-thalassemia should be performed in all suspected patients because it is the gold standard for this diagnosis. However, the financial limitations of the health systems of poorer populations make us look for alternatives to optimize this diagnosis, to prioritize the investigation of individuals who may have impairment of a larger number of α-globin genes, and who have greater clinical impacts. Besides the excessive cost of the molecular technique, it is important to emphasize the need for technical conditions and qualified personnel for conducting this analysis, and all other tests used for the preliminary identification of thalassemia carriers. If we can distinguish individuals who have two or more α-genes deleted using the proposed method, this can generate savings for the health systems in lower and middle-income countries. 

The model developed with the training set showed excellent accuracy (AUC = 0.927, accuracy of 85.1%). It also demonstrated good discriminatory power in the validation set (AUC = 0.902, accuracy of 85.6%) and in the total sample (n = 294). This model also presented high sensitivity and NPV, which allows us to ensure, with a higher level of confidence, that the individual who has a negative result has a minor OR of having two or more deletions related to α-thalassemia. In our total sample, 63.6% (187/294) of the patients would not need an immediate PCR for the most frequent α-thalassemia deletions. This contributes to the decision-making process in the diagnostic investigation, benefiting both the physician and the patient. The physician will have a simple tool that will increase the molecular analysis pre-test probability, based on the most easily available screening tests. Hence, using the BLR model, almost two-thirds of the patients will be spared the exam, which will directly impact the health costs and, additionally, diminish the psychological stress related to the test. 

Those patients showing laboratory characteristics strongly related to α-thalassemia will be forwarded for PCR analysis. The model has a high NPV. It indicates that patients with OR ≤ 0.4 have a high chance of preserving three or four functioning α-genes, excluding a clinically significant disease. It is important to note that the results could be different, and, in reality, better when applied to the general population since the model originated from a specialized laboratory in which patients were sent for analysis due to the suspicion of α-thalassemia or the presence of microcytosis in a previously performed blood count.

We think that another advantage of the model is its strong biological justification. The theoretical basis of the proposed model involves the knowledge that the MCV value and the percentage of Hb A_2_ gradually decrease with the increasing number of α-genes mutated, which results in a consequent reduction in the synthesis of α-globin chains [2,36,37]. Due to the unbalanced synthesis of the α-globin chains, there is a deficiency in the production of hemoglobin per cell and, consequently, a decrease in MCV [38]. Simultaneously, the production of Hb A_2_ decreases due to the decline of α_2_δ_2_ tetramers formation [39]. Since the δ-globin chains have a higher positive charge than the β-globin chains, the Hb A_2_ production is usually hampered relative to the Hb A_1_ [19,39]. In the milder types of α-thalassemia (α^+^ heterozygous, α^+^ homozygous, and α^0^ heterozygous), there is an overlapping percentage of Hb A_2_ among these genotypes, which does not always allow a clear distinction between the distinct types of α-thalassemia. The reduced level of Hb A_2_ is usually more evident in patients with HbH disease [19,38,40]. Serum ferritin tends to increase with the number of the α-globin genes mutated. Some older patients with HbH disease can have iron overload, due to increased iron absorption in the intestine, independent of the transfusion received [2,41,42]. The model shows that there is a greater probability that the patients with two or more deletions have the smaller MCV standardized by age, the smaller percentage of Hb A_2_, and the greater serum ferritin. 

Among the possible limitations of our study, we can mention that the BRL model needs to be evaluated in another cohort, such as an interlaboratory cohort. It would be interesting to validate the model in a larger number of patients to prove its efficacy and reproducibility in distinct regions, populations, and in different age and ethnic groups. Furthermore, our model has not been evaluated for non-deletional α-thalassemia and other less frequent α-globin gene deletions. As a result, we cannot affirm that it would be informative under these conditions.

Our model was built to increase the molecular analysis pre-test probability for two or more deletions. We also tried to apply this model to screen patients with one α-globin gene deletion. However, the test performance did not distinguish the individuals without α-thalassemia from those with a deletion in an α-globin gene. Thus, the proposed BLR model cannot identify patients with only one α-globin gene deleted (silent carrier). It is known that patients suffering from a single gene deletion have minimal or absent laboratory changes, due to a decreased synthesis of α-chains. Hence, they have very similar laboratory tests to individuals who have no alteration in α-globin genes. 

Although the patients with α^+^-thalassemia are asymptomatic, they can generate children with HbH disease in countries where there is a high prevalence of α^0^-thalassemia [2,43,44,45], and in countries where the immigration of the Asian population has increased in recent years. It is important to emphasize that couples with microcytosis and/or anemia who claim to be a parent, as well as pregnant women, should be assessed for mutations related to α-thalassemia, regardless of the outcome of our model. That is because there is a risk of producing a child with HbH disease, and risks of complications in pregnancy due to α-thalassemia, which can be associated with a common iron deficiency in pregnancy (fetal growth restriction, preterm birth, and low birth weight). In such cases, the cause of anemia or microcytosis should be identified correctly and early for appropriate intervention and genetic counseling [46,47].

## 5. Conclusions

This BLR model will help in the decision-making process to indicate molecular testing for α-thalassemia for patients who have a higher OR of presenting α-thalassemia traits (with two α-genes functioning) or intermedia (HbH disease, with one α-gene functioning). The model is easy to apply, requiring data commonly obtained during the investigation of microcytic and hypochromic anemias. As with other mathematical formulas, it is recommended to validate it in larger cohorts and different populations. The model is useful as a decision tool, especially in regions with limited healthcare resources, and particularly in developing countries. 

## Figures and Tables

**Table 1:** Univariate analysis of the variables tested for the BLR model building (n = 134).

Variables	OR	95% Confidence Interval for OR		*p*-Value
		Lower Bound	Upper Bound	
RCC	8.813	3.365	23.087	**<0.001**
Hemoglobin	0.807	0.598	1.089	**0.161**
Hematocrit	0.986	0.889	1.095	0.797
MCV	0.860	0.804	0.919	**<0.001**
MCV standardized by age	0.814	0.751	0.883	**<0.001**
MCH	0.618	0.508	0.752	**<0.001**
MCHC	0.596	0.444	0.800	**0.001**
RDW	1.040	0.909	1.189	0.567
Reticulocyte Count	1.005	0.995	1.015	0.343
% Hb A_1_	1.041	0.989	1.097	**0.124**
% Hb A_2_	0.393	0.176	0.878	**0.023**
% Hb F	0.754	0.471	1.207	0.240
Serum Iron	1.019	1.006	1.032	**0.003**
TIBC	0.996	0.990	1.003	0.264
Serum Ferritin	1.007	0.999	1.014	**0.074**
Transferrin Saturation	1.002	0.992	1.012	0.688
LDH	0.997	0.993	1.000	**0.085**
Indirect Bilirubin	1.737	0.300	10.043	0.538

**Table 2:** Parsimonious BLR model for prediction of genotypes with 2 or more α-globin genes deletions.

Two or More α-Globin Gene Deletions	B	*p*-Value	OR (Exp(B))	IC95
Intercept	21.905	<0.001		
MCV standardized by age	−0.284	<0.001	0.753	0.676–0.839
Serum Ferritin	0.024	<0.001	1.024	1.011–1.037
% Hb A_2_	−1.142	0.006	0.319	0.142–0.715

**Table 3:** Summary of the BLR Model for prediction of genotypes with 2 or more α-globin gene deletions.

	Prediction Equation
**OR**	=exp [21.905 − 0.284 (MCV standardized by age(fL)) + 0.024 (serum ferritin(ng/mL)) − 1.142 (Hb A_2_(%))]
**Probability** (*P* α-thal with 2 or more α-globin genes deletions)	=OR/(1 + OR)

**Table 4:** Basic population characteristics and key BLR model variables.

	Training Set Frequency (%)/Median (IQR)/Mean (SE) n = 134	Validation Set Frequency (%)/Median (IQR)/Mean (SE) n = 160	*p*-Value
**Age (years)**	9.5 (10.3)	8.5 (10.1)	0.551 *
**Gender**			
**Male**	67 (50.0%)	86 (53.8%)	0.559 ***
**Female**	67 (50.0%)	74 (46.2%)	
**The Genotype of the α-Globin Gene**			
**αα/αα**	53 (39.6%)	69 (43.1%)	0.801 ***
**-α^3.7^/αα**	46 (34.3%)	50 (31.3%)	
**-α^3.7^/-α^3.7^**	33 (24.6%)	41 (25.6%)	
**-α^3.7^/- ^SEA^**	1 (0.7%)	—	
**αα/- ^SEA^**	1 (0.7%)	—	
**Variables of the BLR model**			
**MCV standardized by age (fL)**	76.3 (± 0.71)	75.6 (± 0.63)	0.470 **
**Serum Ferritin (ng/mL)**	32.1 (35.7)	31.2 (36.2)	0.726 *
**Hb A_2_ (%)**	2.8 (0.4)	2.8 (0.4)	0.514 *

* Mann-Whitney Test; ** Student’s *t*-test; *** Fisher’s Exact Test.

**Table 5:** Performance evaluation of the proposed model.

	Training Set		Validation Set		Total Sample	
	(n = 134)		(n = 160)		(n = 294)	
		**C.I. (95%)**		**C.I. (95%)**		**C.I. (95%)**
**Sensitivity**	**88.6%**	73.3–96.8%	**95.1%**	83.5–99.4%	**92.1%**	83.6–97.1%
**Specificity**	**83.8%**	75.1–90.5%	**82.4%**	74.3–88.7%	**83.0%**	77.4–87.8%
**Positive Likelihood Ratio**	**5.48**	3.45 -8.72	**5.39**	3.63–8.00	**5.43**	4.02–7.33
**Negative Likelihood Ratio**	**0.14**	0.05–0.34	**0.06**	0.02–0.23	**0.10**	0.04–0.21
**Disease Prevalence**	**26.1%**	18.9–34.4%	**25.6%**	19.1–33.1%	**25.9%**	20.9–31.3%
**Positive Predictive Value**	**66.0%**	54.9–75.5%	**65.0%**	55.6–73.4%	**65.4%**	58.3–71.9%
**Negative Predictive Value**	**95.4%**	89.2–98.1%	**98.0%**	92.7–99.5%	**96.8%**	93.3–98.5%
**Cut-off Point Accuracy**	**85.1%**	77.9–90.6%	**85.6%**	79.2–90.7%	**85.4%**	80.8–89.2%
**Global Accuracy (AUC)**	**0.927**	0.883–0.970	**0.902**	0.853–0.950	**0.914**	0.881–0.947

## Data Availability

The data used in this study are available as Supplementary Materials (Tables S1–S3 and Excel worksheet S4).

## Supplementary Materials

The following supporting information can be downloaded at: https://www.mdpi.com/article/10.3390/diagnostics12123008/s1, Table S1: 2 × 2 Table Comparing BRL Model Prediction Group to Multiplex PCR Results (Training Set); Table S2: 2 × 2 Table Comparing BRL Model Prediction Group to Multiplex PCR Results (Validation Set); Table S3: 2 × 2 Table Comparing BRL Model Prediction Group to Multiplex PCR Results (Total Sample) and Excel worksheet S4: Table for Calculating the Standardized MCV (fL) and Table for Calculating the Prediction of Logistic Regression Model.
