# Supplementary material for: Novel Decision Tool for More Severe α-Thalassemia Genotypes Screening with Functional Loss of Two or More α-Globin Genes: A Diagnostic Test Study
Source: Diagnostics (Basel). 2022 Dec 1;12(12):3008. doi: 10.3390/diagnostics12123008 (PMC9777031; doi:10.3390/diagnostics12123008)
Supplement: Supplementary file 1 [file diagnostics-12-03008-s001.zip › Supplementary Materials Tables S1-S3.pdf]

Supplementary Materials

Table S1. 2 × 2 Table Comparing BRL Model Prediction Group to Multiplex PCR Results (Training Set)

	Multiplex PCR Result (Training Set)			
**Odds Ratio Algorithm**	**Prediction**	**Present**	**Absent**	**Total**
OR = exp [21.905 – 0.284 (MCV standardized by age(fL) + 0.024 (serum ferritin(ng/mL) - 1.142 (Hb A2(%)]	Positive	31	16	47
	Negative	4	83	87
	Total	35	99	134

Table S2. 2 × 2 Table Comparing BRL Model Prediction Group to Multiplex PCR Results (Validation Set)

	Multiplex PCR Result (Validation Set)			
**Odds Ratio Algorithm**	**Prediction**	**Present**	**Absent**	**Total**
OR = exp [21.905 – 0.284 (MCV standardized by age(fL) + 0.024 (serum ferritin(ng/mL) - 1.142 (Hb A2(%)]	Positive	39	21	60
	Negative	2	98	100
	Total	41	119	160

Table S3. 2 × 2 Table Comparing BRL Model Prediction Group to Multiplex PCR Results (Total Sample)

	Multiplex PCR Result (Total Set)			
**Odds Ratio Algorithm**	**Prediction**	**Present**	**Absent**	**Total**
OR = exp [21.905 – 0.284 (MCV standardized by age(fL) + 0.024 (serum ferritin(ng/mL) - 1.142 (Hb A2(%)]	Positive	70	37	107
	Negative	6	181	187
	Total	76	218	294
